# Needs of and Services for Grandparents Raising Grandchildren: Regional, National, and International Perspectives

**DOI:** 10.1093/geroni/igaa057.2056

**Published:** 2020-12-16

**Authors:** Youjung Lee, Deborah Whitley

## Abstract

Grandparents raising grandchildren build strong foundations for their grandchildren. Despite grandparents’ significant contributions to their grandchildren’s future and society in general, there is a limited understanding of the unique needs and service utilization of grandparents raising grandchildren in various contexts. This symposium is focused on the needs of and services for the grandparent population at the regional, national, and international levels. Stucki will present findings from an examination of types and locally available services for grandparents raising grandchildren in Appalachia by sub-region. Musil and colleagues will discuss the service need utilization and unmet service needs of a nationwide sample of 284 grandmothers living with/ raising grandchildren and the relationships between service use/need and resilience, resourcefulness, perceived stress, reward, and appraisals of their current living environment for themselves and their grandchildren. Lastly, Lee will describe research findings from her comparative transnational research on needs and experiences of grandparents raising grandchildren in Malawi (n=29), South Korea (n=23), and the U.S. (n=23). Unique needs and cultural interpretation of intergenerational caregiving in each country will be presented. The symposium discussion will address diverse needs of grandparents raising grandchildren and strategies to meet those needs at regional, national, and international levels. Grandparents as Caregivers Interest Group Sponsored Symposium.

